# Natural Deep Eutectic Solvent-Based Microwave-Assisted Extraction of Total Flavonoid Compounds from Spent Sweet Potato (*Ipomoea batatas* L.) Leaves: Optimization and Antioxidant and Bacteriostatic Activity

**DOI:** 10.3390/molecules27185985

**Published:** 2022-09-14

**Authors:** Yuqin Zhang, Shiquan Bian, Jing Hu, Gang Liu, Shouhua Peng, Hongjiang Chen, Zhenying Jiang, Tongyong Wang, Quan Ye, Haibo Zhu

**Affiliations:** 1 Weihai Academy of Agricultural Sciences, Weihai 264200, China; 2 Key Laboratory of Rice Genetic Breeding of Anhui Province, Rice Research Institute, Anhui Academy of Agricultural Sciences, Hefei 230031, China; 3 Weihai Comprehensive Agricultural Law Enforcement Detachment, Weihai 264200, China; 4 Integrated Agricultural Service Center of Xujia Town, Rushan 264500, China

**Keywords:** natural deep eutectic solvents, response surface, spent sweet potato leaves, antioxidant activity

## Abstract

Natural deep eutectic solvents (NADESs) coupled with microwave-assisted extraction (MAE) were applied to extract total flavonoid compounds from spent sweet potato (*Ipomoea batatas* L.) leaves. In this study, ten different NADESs were successfully synthesized for the MAE. Based on single-factor experiments, the response surface methodology (RSM) was applied, and the microwave power, extraction temperature, extraction time, and solid–liquid ratio were further evaluated in order to optimize the yields of total flavonoid compounds. Besides, the extracts were recovered by macroporous resin for the biological activity detection of flavonoid compounds. As a result, NADES-2, synthesized by choline chloride and malic acid (molar ratio 1:2), exhibited the highest extraction yield. After that, the NADES-2-based MAE process was optimized and the optimal conditions were as follows: microwave power of 470 W, extraction temperature of 54 °C, extraction time of 21 min, and solid–liquid ratio of 70 mg/mL. The extraction yield (40.21 ± 0.23 mg rutin equivalents/g sweet potato leaves) of the model validation experiment was demonstrated to be in accordance with the predicted value (40.49 mg rutin equivalents/g sweet potato leaves). In addition, flavonoid compounds were efficiently recovered from NADES-extracts with a high recovery yield (>85%) using AB-8 macroporous resin. The bioactivity experiments in vitro confirmed that total flavonoid compounds had good DPPH and O_2_^−^· radical-scavenging activity, as well as inhibitory effects on *E. coli*, *S. aureus*, *E. carotovora*, and *B. subtilis*. In conclusion, this study provides a green and efficient method to extract flavonoid compounds from spent sweet potato leaves, providing technical support for the development and utilization of sweet potato leaves’ waste.

## 1. Introduction

Sweet potato leaves refer to the tender leaves at the top of aboveground seedlings after sweet potato matures in autumn. They are often neglected by-products in the process of sweet potato planting [1,2,3]. Many studies have found that sweet potato leaves are rich in polyphenols, proteins, vitamins, minerals, and other nutrients, with high nutritional value [1,4,5,6,7]. They have a variety of health care effects such as improving immunity, antioxidation, bacteriostasis, and promoting metabolism [2,5,8,9,10]. Sweet potato leaves have been regarded as a new type of vegetable and have come to people’s table. However, the development and utilization of sweet potato leaves in China is very limited. Most of the sweet potato leaves are used as livestock feed or are directly abandoned, resulting in a huge waste of resources. The output of sweet potato in China accounts for 80% of the world, while the utilization rate of sweet potato leaves is less than 10% [11]. The main reasons are that the extraction process of sweet potato leaves is immature and there is a lack of research on its properties. Therefore, it is of great significance to develop the extraction process of sweet potato leaves and test the properties of the extracts.

How to extract flavonoid compounds from spent sweet potato leaves efficiently and organically has attracted much attention. Traditional organic solvents, such as ethanol, chloroform, and ethyl acetate, are widely used as extractants in the pharmaceutical, food, cosmetics, and other industries [12,13,14]. However, the extensive use of organic solvents will pollute the environment and lead to solvent residues. Therefore, it is very important to choose a natural and green extraction solvent. Natural deep eutectic solvents (NADESs) are a series of solvents with physical properties similar to ionic liquids (ILs) [15,16,17]. At present, NADESs composed of choline, urea, organic acid, and sugar have been reported [18,19]. Compared with ILS and traditional organic solvents, NADES has economic and environmental advantages, such as biodegradability, recyclability, low-cost, simple-preparation method, and so on [15,18,19,20,21,22,23]. In addition, NADES has strong solubilization ability for both non-polar and polar compounds, causing high extraction efficiency for bioactive components [24,25]. At the same time, NADES can dissolve macromolecules.

Therefore, NADESs as solvents to extract valuable bioactive components have great development prospects in the food, cosmetics, and pharmaceutical industries. It has been reported that NADESs replace traditional solvents to extract phenolic secondary metabolites in safflower, total flavonoids in Sophora japonica flowers, alkaloids in Salvia miltiorrhiza, and polyphenols in seabuckthorn seed meal [15,23,26,27], but there is no report that NADES is used to extract flavonoid compounds from spent sweet potato leaves.

In this study, spent sweet potato leaves were used as raw materials to extract total flavonoid compounds by microwave-assisted NADES. The optimal extraction process was determined through single-factor test and response surface optimization. The antioxidant activity and bacteriostatic activity of flavonoid compounds from spent sweet potato leaves were further investigated, which can provide a theoretical basis for the development and utilization of spent sweet potato leaves in the pharmaceutical, food, cosmetics, and other industries.

## 2. Materials and Methods

### 2.1. Chemicals and Reagents

Choline chloride, betaine, L-proline, lactic acid, malic acid, glacial acetic acid, glucose, fructose, xylitol, glycerol, ethylene glycol, 1,4-butanediol, urea, and levulinic acid for the synthesis of NADESs were purchased from Aladdin Reagent Company (Shanghai, China). The standard compound rutin (purity > 99.5%) was purchased from Shanghai Yuanye Bio-Technology Co., Ltd. (Shanghai, China). HPLC-grade acetic acid and methanol were all purchased from Fisher Scientific (Waltham, MA, USA). Macroporous resins (D101, AB-8, and DM130) were purchased from Anhui Sanxing Resin Technology Co., Ltd. (Anhui, China). 1,1-diphenyl-2-picrylhydrazyl (DPPH) was purchased from Sigma-Aldrich Chemical Co., Ltd. (St. Louis, MO, USA). Other experimental supplies were from Weihai Academy of Agricultural Sciences (Weihai, China). All other reagents and chemicals were of analytical grade.

### 2.2. Preparation and Synthesis of NADESs

NADESs were synthesized easily following the stirring and heating method described by different works in the literature [19,21]. A total of ten different types of NADESs were prepared by stirring a hydrogen bond acceptor (choline chloride) with hydrogen bond donors (lactic acid, malic acid, glacial acetic acid, and so on) at a certain molar ratio. NADESs, a series of colorless transparent homogeneous liquids, were obtained at 80 °C for 4–6 h without any visual precipitate. The prepared NADESs were stored at 4 °C for subsequent extraction. The abbreviations, compositions, and molar ratio of NADESs used in this study are all shown in Table 1.

### 2.3. Materials

Spent sweet potato (Weishu No.6) leaves (SPLs) were obtained from the Weihai Academy of Agricultural Sciences (Weihai, Shandong, China). They were first freeze-dried in a freeze-dryer (FD-2C, Bilang Instrument Co., Ltd., Shanghai, China) at −60 °C for 48 h and then ground to a fine powder in a small grinder (DE-2000, Zhejiang Rhodiola Industry and Trade Co., Ltd., Jinhua, China). The appropriate powder of 60 mesh was selected for further extractions and stored in the dark at 4 °C.

### 2.4. Microwave-Assisted Extraction of SPLs

The microwave-assisted extraction (MAE) process was performed in the microwave extractor (MAS-Ⅱ, Xinyi Microwave Chemical Technology Co., Ltd., Shanghai, China). In order to obtain flavonoids from SPLs, 0.1 g of sample powder was added into a 20 mL round-bottom flask and mixed with a certain amount of NADES systems (water content in extraction systems: 30%, *w*/*w*) according to the selected solid–liquid ratios (20, 40, 60, 80, and 100 mg/mL). Then, the round-bottom flask was placed in the microwave extractor with the condensing device, and the mixture was extracted for a certain amount of time (10, 20, 30, 40, and 50 min) at a certain temperature (30, 40, 50, 60, and 70 °C) and microwave power (100, 200, 300, 400, and 500 W). After the extraction, the solution was transferred and centrifuged (Centrifuge 5804, Eppendorf AG, Hamburg, Germany) for 10 min (8000 G, 4 °C) to obtain the supernatant. The liquid samples collected were stored at 4 °C for the further determination of total flavonoid compounds.

### 2.5. Assessment of Total Flavonoid Compounds from SPLs

The assessment of total flavonoid compounds (TFCs) from SPLs using NADESs was carried out according to Namazi et al. [28]. Ten-fold-diluted flavonoid extract (50 μL) was added into 96-well plates and mixed with 10 μL of sodium nitrite solution (5%, *w*/*v*) for 5 min at room temperature. Subsequently, 10 μL of aluminum chloride (10%, *w*/*v*) was added and thoroughly mixed for 1 min. Ultimately, 100 μL of NaOH solution (0.5 M) was added and incubated for 15 min. After gentle mixing, the reaction systems were measured for absorbance at 510 nm. The yields of total flavonoid compounds were expressed as milligrams of rutin equivalents per gram of SPLs (mg RE/g SPLs). The standard curve equation of rutin was Y = 0.00847X + 0.0179 (R^2^ = 0.9995, n = 7), with a linear range of 10~100 μg/mL.

### 2.6. Single-Factor Experimental Design

According to the results of previous studies [22], the main factors affecting the efficiency of microwave-assisted extraction, including extraction time (10, 20, 30, 40, and 50 min), extraction temperature (30, 40, 50, 60, and 70 °C), microwave power (100, 200, 300, 400, and 500 W), and solid–liquid ratios (20, 40, 60, 80, and 100 mg/mL), were tested by single-factor experiments. The yields of flavonoid compounds from SPLs were calculated by changing only one main influencing factor and keeping the other conditions unchanged.

### 2.7. Optimal Design of NADES-Based Microwave-Assisted Extraction

Response surface methodology (RSM) was conducted for the best extraction conditions by Design-Expert Ver. 8.1.5 (Stat-Ease Inc., Minneapolis, MN, USA). Based on the Box–Behnken design (BBD), four main variables, including microwave power (X_1_, W), extraction temperature (X_2_, °C), extraction time (X_3_, min), and solid–liquid ratio (X_4_, mg/mL), and the interactions among them were estimated (Table 2). As the response of the design experiments, the yields of total flavonoid compounds from SPLs were also measured accurately. The multiple regression analysis was used to structure a prediction model by correlating the measured responses with independent variables.

### 2.8. HPLC Analysis of Flavonoid Compounds from SPLs Using NADESs

A simple qualitative analysis of the flavonoid compounds from SPLs using NADESs was carried out by HPLC. Agilent C18 column (250 mm × 4.6 mm, 5 μm) was selected in this study. The mobile phase was methanol (A) and 0.5% acetic acid aqueous solution (B), and gradient elution was carried out as follows: 0–10 min, 25% A; 10–20 min, 35% A; 20–30 min, 40% A; 30–60 min, 50% A. The injection volume was 10 μL and the column temperature was 28 °C. The volume flow rate and the detection wavelength were 1.0 mL/min and 360 nm, respectively.

### 2.9. Separation and Recovery of Flavonoids by Macroporous Resins

Macroporous resins D101, AB-8, and DM130 (Table 3) were tested to separate and obtain flavonoid compounds from the NADES-extract mixture, respectively. The NADES-extract mixture (5 mL) was loaded in a 50 mL syringe filled containing 10 g of the pretreated macroporous resins. The elution procedure was as follows: washing with 50 mL of deionized water, followed by elution with 20 mL of 50% methanol and 20 mL of 100% methanol. The flow rates of deionized water and methanol were 2 mL/min and 1 mL/min, respectively. Methanol elution phases were fully collected and dried with a vacuum evaporator (RE-6000A, Shanghai, China) for bioactivity assay. The rate of recovery (%) was calculated according to the following equation:

Recovery rate (%) = C_m_ × V_m_/C_e_ × V_e_

where C_m_ are the concentrations of total flavonoid compounds in methanol elution phases, V_m_ are the total volumes of methanol elution phases, C_e_ are the concentrations of total flavonoid compounds in the NADES-extract mixture, and V_e_ are the volumes of the NADES-extract mixture.

### 2.10. Assessment of Antioxidant Activity In Vitro

Total flavonoid compounds extracted under the optimal extraction conditions in methanol elution phases were collected and measured for antioxidant activity, including DPPH and O_2_^−^·assays. In order to intuitively reflect the antioxidant activity of the target compounds, butylated hydroxytoluene (BHT) and vitamin E (VE) were used as the positive controls.

#### 2.10.1. DPPH Assay

The determination of the DPPH radical scavenging rate of total flavonoid compounds was carried out according to the previous methods [29]. The solution of total flavonoid compounds (50, 100, 150, 200, 250, and 300 μg/mL; 50 μL) was added into 150 μL of DPPH solution (0.2 mM) freshly prepared in methanol. After fully mixing, the reaction system was reacted at 30 °C in the dark for 30 min. The absorbance value of the solution system was determined at 517 nm. Similarly, BHT and VE with the same concentrations in methanol solution were carried out according to the above processes. The DPPH radical scavenging rate was calculated according to the following formula:

DPPH radical scavenging rate (%) = (A_b_ − A_s_)/A_b_

where A_b_ is the absorbance value of blank sample and A_s_ is the absorbance value of total flavonoid compounds from SPLs.

#### 2.10.2. O_2_^−^ Assay

First, 100 μL of Tris-HCl buffer (0.05 M, pH 8.2) was preheated at 25 °C for 20 min. Then, total flavonoid compounds (50, 100, 150, 200, 250, and 300 μg/mL; 30 μL) and 20 μL of pyrogallol solution (0.025 M) were added into the Tris-HCl buffer. After fully mixing, the reaction system was reacted at 25 °C for 5 min. Then, 50 μL of HCL was added to terminate the reaction and the absorbance value was determined at 299 nm. The O_2_^−^· radical scavenging rate was calculated according to the following formula:

O_2_^−^· radical scavenging rate (%) = (A_b_ − A_s_)/A_b_

where A_b_ is the absorbance value of blank sample and A_s_ is the absorbance value of total flavonoid compounds from SPLs.

### 2.11. Assessment of Bacteriostatic Activity

According to Tosi et al. [30], the bacteriostatic activity of total flavonoid compounds from SPLs was evaluated using the plate suppression method. *Escherichia coli*, *Staphylococcus aureus*, *Bacillus subtilis*, *Pseudomonas aeruginosa*, and *Erwinia carotovora* were selected as the inhibitory bacterial strains. The bacterial suspension (10^6^ CFU/mL, 200 μL) was inoculated into 20 mL MH agar medium. Three holes (5 mm) were drilled evenly on each MH agar medium with a sterile puncher. The penicillin–streptomycin solution (1000 U/mL, 10 μL), DMSO solution (10 μL), and 200 μg/mL total flavonoid compounds dissolved in DMSO solution (10 μL) were added into one hole, respectively. The MH agar mediums were all incubated at 37 °C for 36 h, and then the diameter of the inhibition zone was measured.

## 3. Results and Discussion

### 3.1. Optimization of NADES Systems

**Figure 1:**
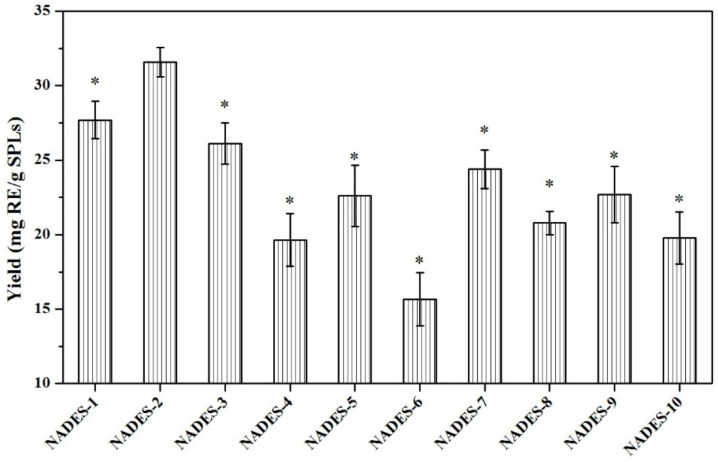
**Extraction yields of total flavonoid compounds from SPLs.** Extraction conditions: 0.1 g of SPLs in 1 mL of NADES systems (30% water, *w*/*w*) by MAE (200 W) at 50 °C for 20 min. Yields of total flavonoid compounds that were significantly different from NADES-2 were indicated with * (*p* < 0.05).

In the extraction process of total flavonoids from plant materials, the compositions of NADES have been reported to impact on the extraction efficiency [22]. In this study, to find the best NADES system, different types of NADESs (Table 4) were synthesized and used for the extraction of total flavonoids from SPLs. As shown in Figure 1, the quantitative results indicated that all carboxylic-acid-based NADESs (NADES-1, NADES-2, and NADES-3) had great extraction yields of total flavonoid compounds from SPLs. However, sugar-based NADESs (NADES-4, NADES-5, and NADES-6) with high viscosity hampered mass transfer and diffusion of compounds, which led to the low yields of total flavonoid compounds. These results are in agreement with previous findings reported by Shang et al. [19]. In addition, we found that total flavonoid compounds were more difficult to extract by alcohol-based NADESs (NADES-7, NADES-8, and NADES-9) and urea-based NADES (NADES-10). This phenomenon may be due to the hydrogen bond, van der Waals, and electrostatic interactions between the constitutes, which restrict their application in the extraction of bioactive compounds.

The difference significance analysis showed that NADES-2 composed of choline chloride-malic acid at ratio of 1:2 had the highest yields of total flavonoid compounds (31.56 ± 0.98 mg RE/g SPLs), significantly higher than other NADESs (*p* < 0.05). Hence, NADES-2 was used in the further experiments.

### 3.2. NADES-MAE Optimization by Single-Factor Experiments

**Figure 2:**
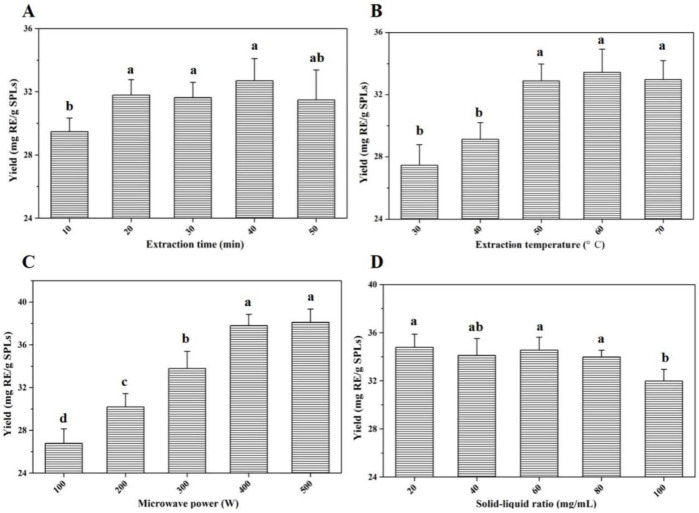
**Extraction yields of total flavonoid compounds from SPLs with different influencing factors.** Invariant extraction conditions: 200 W, 50 °C, and 100 mg/mL (**A**); 200 W, 20 min, and 100 mg/mL (**B**); 20 min, 50 °C, and 100 mg/mL (**C**); 200 W, 20 min, and 50 °C (**D**). Data sharing different letters were significantly different (*p* < 0.05).

Based on the main influencing factors in the extraction process, single-factor optimization experiments were carried out in this section. The effects of extraction time (min), extraction temperature (°C), microwave power (W), and solid–liquid ratio (mg/mL) on yields of total flavonoid compounds were evaluated, respectively. As shown in Figure 2A, under the same extraction conditions, the yields of total flavonoid compounds were not significantly affected by the changes in time. When the extraction time was 20 min, the yield basically reached the peak value. In the range of 30~50 °C, the yields increased slowly with the increase in extraction temperature, and reached the maximum value at 50 °C. As the temperature continued to rise, there was no significant change in the extraction yields of total flavonoids (Figure 2B). Similarly, the quantitative results clearly indicated that microwave power of 400 W and a solid–liquid ratio of 80 mg/mL were conducive to the extraction of total flavonoids from SPLs (Figure 2C,D).

### 3.3. NADES-MAE Optimization by BBD

In order to obtain the best extraction parameters for NADES-MAE, microwave power (X_1_, W), extraction temperature (X_2_, °C), extraction time (X_3_, min), and solid–liquid ratio (X_4_, mg/mL) were selected as the main influencing factors in the RSM. As shown in Table 4, the design combination of influencing factors and the corresponding extraction yields of total flavonoid compounds from SPLs were evaluated. Based on the Box–Behnken design, a theoretical prediction model was obtained and the second-order polynomial equation of this model was as follows:

Y = 40.49 + 1.59X_1_ + 0.20X_2_ + 0.14X_3_ − 0.53X_4_ + 0.14X_1_X_2_ − 0.018X_1_X_3_ − 0.14X_1_X_4_ − 0.053X_2_X_3_ + 0.12X_2_X_4_ + 0.015X_3_X_4_ − 1.83X_1_^2^ − 0.36X_2_^2^ − 0.54X_3_^2^ − 0.61X_4_^2^.

Table 5 lists the ANOVA results of the model, and the *F*-value of model was 24.61, which implied that this prediction model was significant. Similarly, the *p*-value was <0.0001, indicating that this model was inaccurate with a probability of only 1%. Among the four main influencing factors, X_1_ and X_2_ had significant impacts on the extraction yields of total flavonoid compounds from SPLs (*p* < 0.05). In addition, the “pred R-squared” of 0.7762 was in reasonable agreement with the “adj R-squared” of 0.9219. The above analysis results showed that this model could be used to navigate the design space.

**Figure 3:**
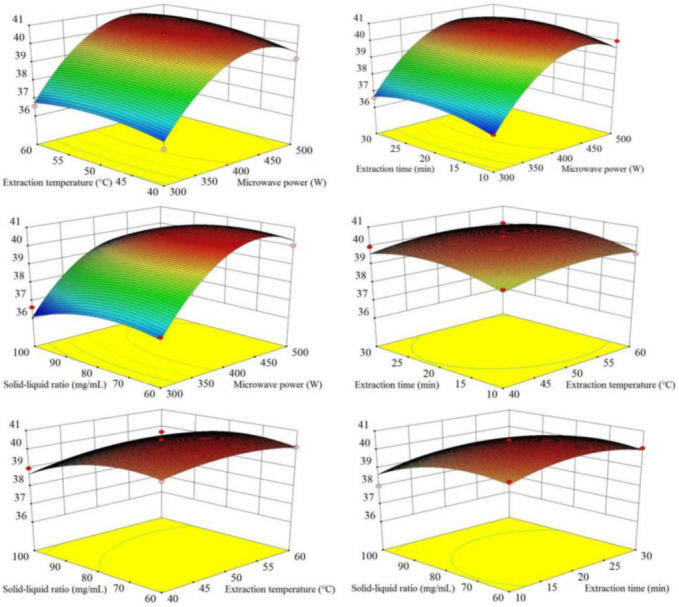
**Three-dimensional (3D) response surface of the response surface design forNADES-MAE optimization.** X_1_, microwave power (W); X_2_, extraction temperature (°C); X_3_, extraction time (min); X_4_, solid–liquid ratio (mg/mL).

The 3D response surface (Figure 3) intuitively showed the interactive relationships of the four variables (X_1_, microwave power; X_2_, extraction temperature; X_3_, extraction time; X_4_, solid–liquid ratio). The results showed that microwave power (X_1_) had the greatest influence, followed by solid–liquid ratio (X_4_), and the influences of extraction temperature and time were slight. The extraction yields of total flavonoid compounds from SPLs increased rapidly with higher extraction power and reached up to an optimal level. In addition, the 3D response surface showed that extraction temperature (X_2_) and extraction time (X_3_) had no interactive effects on extraction yields. These results were consistent with the ANOVA.

Optimization analysis based on the prediction model indicated that the optimal extraction parameters were as follows: microwave power of 470 W, extraction temperature of 54 °C, extraction time of 21 min, and solid–liquid ratio of 70 mg/mL. Under these optimal conditions, the prediction extraction yield was 40.49 mg RE/g SPLs. Through triplicate verification experiments, the actual yield of total flavonoid compounds was 40.21 ± 0.23 mg RE/g SPLs. This verification result also supported that the prediction model obtained was predictable and reproducible.

### 3.4. HPLC Analysis and Recovery of Total Flavonoid Compounds from NADES Systems

**Figure 4:**
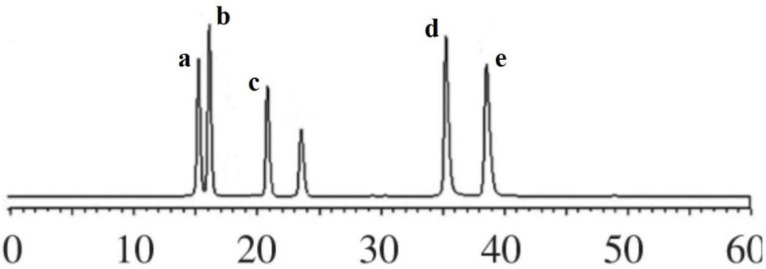
The chromatograms of flavonoid compounds from NADES by HPLC. a, coumarin; b, kaempferol; c, genistein; d, quercetin; e, luteolin.

**Figure 5:**
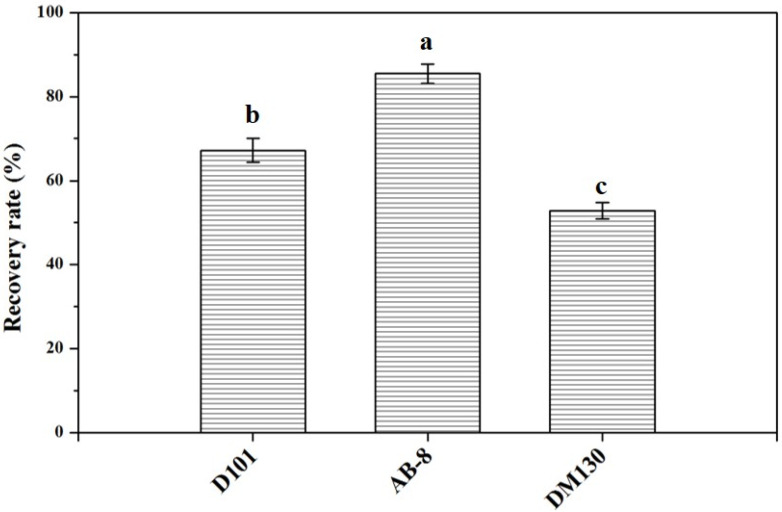
Recovery rates of flavonoid compounds from NADES systems with the three macroporous resins. Data sharing different letters were significantly different (*p* < 0.05).

The chromatograms of flavonoid compounds from NADES by HPLC analysis are shown in Figure 4. By comparing the retention time, five flavonoid compounds were identified, including coumarin, luteolin, quercetin, genistein, and kaempferol. In order to obtain total flavonoid compounds for the further biological activity detections, three different types of macroporous resins (D101, AB-8, and DM130) were tested in this study. The results (Figure 5) showed that macroporous resin AB-8 was the best resin for the adsorption and separation of total flavonoids from SPLs (85.46% ± 2.33%). In addition, resin DM130 exhibited the worst adsorption capacities among the three resins. The rates of recovery may depend on the different characteristics (particle size range, polarity, wet true density, specific surface area, and average aperture) of the three macroporous resins. Based on the above results, macroporous resin AB-8 was selected for the further studies.

### 3.5. Assessment of Antioxidant Activity of Total Flavonoid Compounds In Vitro

#### 3.5.1. DPPH Assay

**Figure 6:**
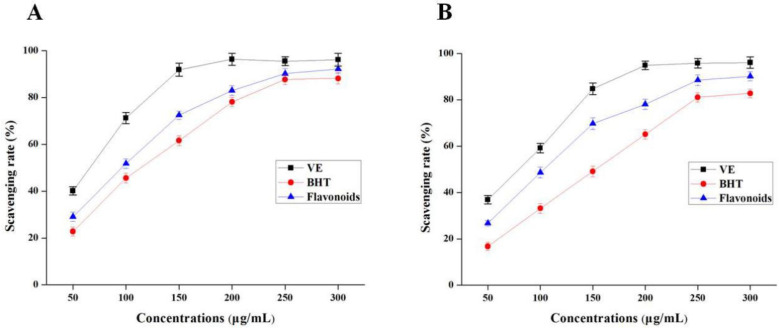
DPPH radical-scavenging activity (**A**) and O_2_^−^• radical -scavenging activity (**B**) of total flavonoid compounds in vitro. BHT, butylated hydroxytoluene; VE, vitamin E.

DPPH radical-scavenging activity is an important index to evaluate antioxidant activity of bioactive compounds in vitro [29]. The DPPH radical-scavenging activity of total flavonoid compounds from SPLs increased with the increase in concentrations from 50 to 300 μg/mL (Figure 6A). Compared with the positive control, the DPPH assay of total flavonoids was stronger than BHT, but weaker than VE at the same concentrations. These results indicated that the total flavonoid compounds from SPLs using NADES systems had excellent DPPH radical-scavenging activity.

#### 3.5.2. O_2_^−^ Assay

The results in Figure 6B show a good dose effect relationship between O_2_^−^· radical-scavenging rate and total flavonoid compounds. The IC_50_ values of total flavonoid compounds, VE, and BHT were 101.2 ± 1.9, 78.9 ± 2.1, and 148.6 ± 0.8 μg/mL, respectively. In the same way, the O_2_^−^· radical-scavenging activity of total flavonoid compounds was lower than that of VE, but much better than BHT.

### 3.6. Assessment of Bacteriostatic Activity of Total Flavonoid Compounds

The results of bacteriostatic activity of total flavonoid compounds are shown in Table 6. As expected, there was no doubt that DMSO solution (the solvent control) had no inhibitory effect on all of the bacterial strains. No obvious inhibition zone was observed on the medium inoculated with *Pseudomonas aeruginosa*, which indicated that total flavonoid compounds from SPLs had no inhibitory effect on *P. aeruginosa*. In addition, the diameters of the inhibition zone of total flavonoid compounds on *Escherichia coli*, *Staphylococcus aureus*, *Erwinia carotovora*, and *Bacillus subtilis* were 4.9 ± 0.8 mm, 13.8 ± 1.4 mm, 12.7 ± 2.2 mm, and 23.6 ± 2.3 mm, respectively. Especially, total flavonoid compounds’ extract from SPLs had the strongest inhibitory effect on *B. subtilis*.

### 3.7. The Correlation between Antioxidant and Bacteriostatic Activities and Contents of the Total Flavonoids Compounds of Spent Sweet Potato Leaves’ Extracts

The correlation analysis results showed that the content of total flavonoids in the extract was significantly correlated with the DPPH assay and O_2_^−^· assay (*p* < 0.05), and the correlation coefficients were 0.916, and 0.911, respectively. In addition, there was a significant positive correlation between bacteriostatic activities and the contents of total flavonoids (*p* < 0.01).

## 4. Conclusions

In this study, natural deep eutectic solvent-based microwave-assisted extraction of total flavonoid compounds from spent sweet potato leaves was used, and NADES-2 synthesized by choline chloride and malic acid (molar ratio 1:2) was the best solvent. The whole extraction process was optimized by single-factor experiments and response surface analysis. The optimum extraction conditions were as follows: microwave power of 470 W, extraction temperature of 54 °C, extraction time of 21 min, and solid–liquid ratio of 70 mg/mL. Under these optimum conditions, the yield of total flavonoid compounds was 40.21 ± 0.23 mg RE/g SPLs. Moreover, the obtained total flavonoid compounds were recovered with three different macroporous adsorption resins, and the recovery rate of macroporous resin AB-8 reached 85.46% ± 2.33%. The experiments of antioxidant activity in vitro showed that total flavonoid compounds had good DPPH and O_2_^−^ radical-scavenging activity. Meanwhile, total flavonoid compounds from SPLs had inhibitory effects on *E. coli*, *S. aureus*, *E. carotovora*, and *B. subtilis*, which provided a theoretical basis for the green and efficient extraction of flavonoid compounds from spent sweet potato leaves and creating greater economic value.

## Figures and Tables

**Table 1:** Different abbreviations, compositions, and molar ratios of NADESs synthesized in this study.

Abbreviation	Hydrogen Bond Acceptor	Hydrogen Bond Donor	Molar Ratio
NADES-1	Choline chloride	Lactic acid	1:2
NADES-2	Choline chloride	Malic acid	1:1
NADES-3	Choline chloride	Glacial acetic acid	1:2
NADES-4	Choline chloride	Glucose	1:1
NADES-5	Choline chloride	Fructose	1:1
NADES-6	Choline chloride	Sucrose	2:1
NADES-7	Choline chloride	Glycerol	1:2
NADES-8	Choline chloride	Ethylene glycol	1:2
NADES-9	Choline chloride	1,4-Butanediol	1:5
NADES-10	Choline chloride	Urea	1:2

**Table 2:** The main variables and levels for BBD in this study.

Variables	Symbols	Coded Levels		
		−1	0	1
Microwave power (W)	X_1_	300	400	500
Extraction temperature (°C)	X_2_	40	50	60
Extraction time (min)	X_3_	10	20	30
Solid–liquid ratio (mg/mL)	X_4_	60	80	100

**Table 3:** The characteristics of three macroporous resins for total flavonoids from SPLs.

Macroporous Resins	Polarity	Particle Size Range (mm)	Specific Surface Area (m^2^/g)	Average Aperture (nm)
D101	Nonpolar	0.3–1.25	500–550	9–10
AB-8	Low-pole	0.3–1.25	480–520	12–16
DM130	Low-pole	0.3–1.25	500–550	9–10

**Table 4:** Results of the Box–Behnken design for extraction optimization.

Run	Variables				Yields (mg RE/g SPLs)
	X_1_ (W)	X_2_ (°C)	X_3_ (min)	X_4_ (mg/mL)	
1	400	50	20	80	40.49
2	500	50	10	80	39.99
3	400	50	10	100	38.01
4	400	60	10	80	39.59
5	300	50	10	80	36.44
6	400	60	20	100	39.67
7	300	60	20	80	36.56
8	500	50	20	60	40.03
9	400	50	20	80	40.52
10	500	60	20	80	40.07
11	400	50	20	80	40.38
12	400	60	30	80	39.99
13	400	50	30	100	38.27
14	500	50	30	80	40.06
15	400	40	20	100	38.98
16	500	40	20	80	39.21
17	400	50	10	60	39.88
18	400	50	20	80	40.51
19	400	50	30	60	40.08
20	500	50	20	100	39.11
21	400	40	10	80	39.33
22	400	60	20	60	40.12
23	300	50	20	60	36.96
24	400	40	30	80	39.94
25	300	50	30	80	36.58
26	300	40	20	80	36.26
27	400	50	20	80	40.54
28	400	40	20	60	39.9
29	300	50	20	100	36.61

X_1_, microwave power (W); X_2_, extraction temperature (°C); X_3_, extraction time (min); X_4_, solid–liquid ratio (mg/mL).

**Table 5:** ANOVA of the regression model for extraction yield.

Source	Sum of Squares	df	Mean Square	*F*-Value	*p*-Value
Model	56.84797	14	4.060569	24.60617	<0.0001
X_1_	30.27363	1	30.27363	183.4517	<0.0001
X_2_	0.472033	1	0.472033	2.86042	0.1129
X_3_	0.2352	1	0.2352	1.425261	0.2524
X_4_	3.328533	1	3.328533	20.17019	0.0005
X_1_X_2_	0.0784	1	0.0784	0.475087	0.5019
X_1_X_3_	0.001225	1	0.001225	0.007423	0.9326
X_1_X_4_	0.081225	1	0.081225	0.492206	0.4944
X_2_X_3_	0.011025	1	0.011025	0.066809	0.7998
X_2_X_4_	0.055225	1	0.055225	0.334652	0.5721
X_3_X_4_	0.0009	1	0.0009	0.005454	0.9422
X_1_^2^	21.65933	1	21.65933	131.2509	<0.0001
X_2_^2^	0.83987	1	0.83987	5.089434	0.0406
X_3_^2^	1.907841	1	1.907841	11.5611	0.0043
X_4_^2^	2.412303	1	2.412303	14.61803	0.0019
Residual	2.310313	14	0.165022		
Lack of Fit	2.294433	10	0.229443	57.79429	0.0007
Pure error	0.01588	4	0.00397		
Cor total	59.15828	28			

**Table 6:** Diameter of the inhibition zone of total flavonoid compounds.

Sample	Diameter of Inhibition Zone (mm)				
	*E. coli*	*S. aureus*	*E. carotovora*	*P. aeruginosa*	*B.subtilis*
Positive control	25.4 ± 2.3	20.5 ± 1.6	31.9 ± 2.9	28.2 ± 1.8	36.5 ± 2.6
Solvent control	-	-	-	-	-
Total flavonoids	4.9 ± 0.8	13.8 ± 1.4	12.7 ± 2.2	-	23.6 ± 2.3

- No inhibition zone.
